# A two-species competition model on a compact metric graph for the invasion and competition of *Aedes Aegypti* and *Aedes Albopictus* mosquitoes in Florida

**DOI:** 10.1007/s00285-026-02421-2

**Published:** 2026-06-23

**Authors:** Canrong Tian, Lan Zou, Shigui Ruan

**Affiliations:** 1 School of Mathematics and Physics, Yancheng Institute of Technology, Yancheng, Jiangsu 224003 P. R. China; 2 School of Mathematical Sciences, Capital Normal University, Beijing, 100048 P. R. China; 3 Department of Mathematics, University of Miami, Coral Gables, FL 33146 USA

**Keywords:** Biological invasion, Competition, *Aedes* mosquitoes, Compact metric graph, Global stability, 35B35, 35K60, 92B05

## Abstract

Motivated by the invasion of *Aedes albopictus* mosquitoes and the interspecific competition between *Aedes aegypti* and *Aedes albopictus* in Florida, we formulate a two-species competition model on a compact metric graph. This model accounts for the species’ ability to inhabit and traverse along the graph’s edges. In the scenario of weak-strong competition, we prove that solutions of the competition model converge uniformly to a semi-positive equilibrium, where either *Aedes albopictus* survives while *Aedes aegypti* goes extinct, or vice versa. Whereas for weak-weak competition, the solutions converge uniformly to a positive equilibrium, enabling the coexistence of both *Aedes aegypti* and *Aedes albopictus*. We conduct numerical simulations on the two-species competition model along Route 441 in Florida, aiming to illustrate the invasion dynamics and competitive interactions of *Aedes aegypti* and *Aedes albopictus*.

## Introduction

*Aedes aegypti* and *Aedes albopictus* are two primary mosquito species implicated in the transmission of viruses causing dengue, yellow fever, chikungunya, Zika, and some other vector-borne illnesses. *Ae. aegypti*, which has a global distribution across tropical and subtropical zones, is an insect strongly linked to human populations and their residential environments. Conversely, *Ae. albopictus* originates from the tropical and subtropical regions of Southeast Asia. As a species highly invasive in nature, *Ae. albopictus* has spread its geographic range to numerous countries and territories in recent decades via international travel and cargo transportation (Hahn et al. 2017; Hawley et al. 1987; Kraemer et al. 2019; Medlock et al. 2015).

**Fig. 1:**
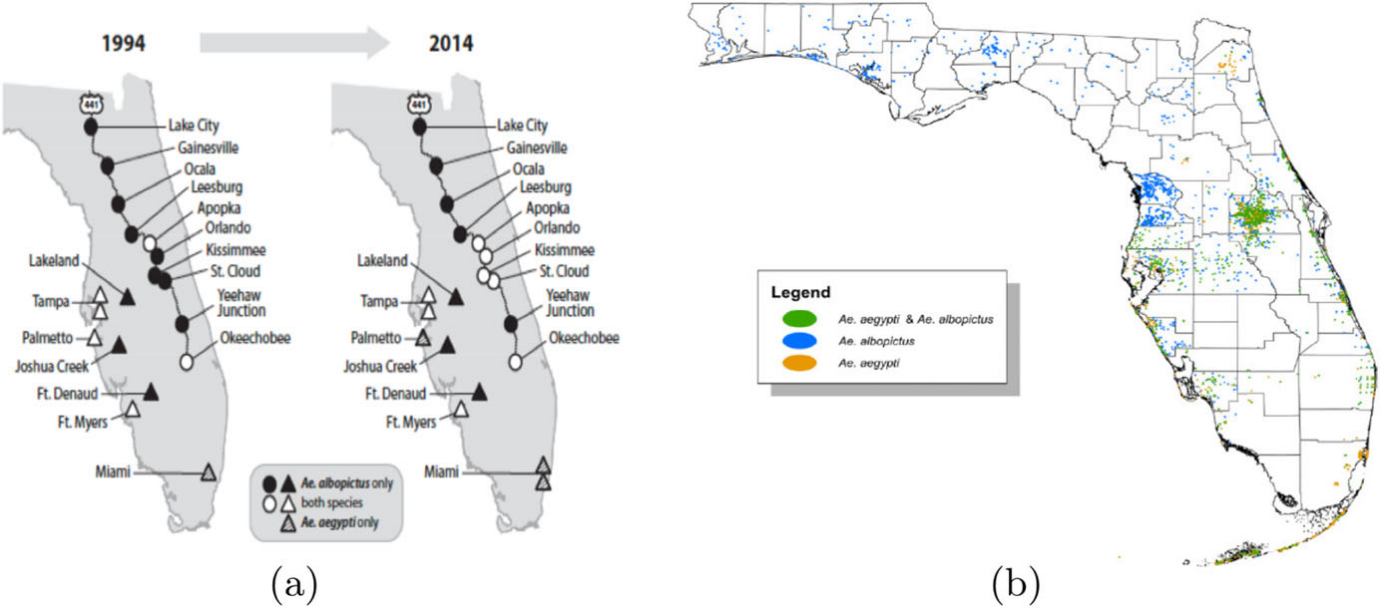
(a) Presence of *Ae. aegypti* and *Ae. albopictus* on Florida State Route 441 between 1994 and 2014, based on collection records of O’meara et al. (1995) and Lounibos et al. (2016), where triangles represent cemeteries and circles represent cities with tire shops. (b) Estimated distribution of *Ae. aegypti* and *Ae. albopictus* in Florida based on positive collections and identifications made from 2011-2018 (Parker et al. 2019)

Kraemer et al. (2019) reported that the spread of *Ae. aegypti* is marked by long-distance importations, while *Ae. albopictus* tends to expand primarily along the peripheries of its existing distribution. In the United States (U.S.), *Ae. albopictus* was first documented in Florida in 1986 (Peacock et al. 1988). By 2008, the species had already established populations in 36 states and continued to expand its range (Enserink 2008), a process facilitated by the interstate transport of used tires (Moore and Mitchell 1997). Annual larval surveillance of *Aedes* mosquitoes in Florida during the early 1990s (O’Meara et al. 1993; O’meara et al. 1995) and in 2013-2014 (Lounibos et al. 2016) revealed their expansion along State Route 441 (Figure 1(a)). A recent survey on the current distribution of *Ae. aegypti* and *Ae. albopictus* in Florida (Parker et al. 2019; Figure 1(b)) illustrates that either *Ae. albopictus* or *Ae. aegypti* were identified in 56 out of 67 counties that contributed data or samples to the research. In the Panhandle region where *Ae. albopictus* was introduced 25 counties reported the presence of *Ae. albopictus* exclusively, while no counties documented *Ae. aegypti*. The survey findings reveal that since 1995, *Ae. aegypti* has reappeared in central Florida where it was previously displaced or undetectable, indicating a potential continuation of its geographic range expansion.

Parker et al. (2019) found competitive interactions between *Ae. aegypti* and *Ae. albopictus*, whereby their larvae compete for limited resources in shared habitats. The spread of invasive species under competitive dynamics represents a pivotal theme in ecological research. Numerous mathematical models formulated via reaction-diffusion equations have been proposed to characterize this phenomenon (Lockwood et al. 2007; Shigesada and Kawasaki 1997; Lou and Zhou 2015). By considering the invasive domain as a free boundary, Tian and Ruan (2018) studied the influence of the advection terms on the asymptotic behavior of the competition between *Ae. aegypti* and *Ae. albopictus* in Florida. Moreover, by replacing the continuous space by a complex network, Liu et al. (2020) have investigated the long-term dynamical behavior of the competition model and shown that the invasive species converges to a positive constant.

However, previous networked models of biological invasion only considered habitats as nodes, without accounting for the fact that species can also inhabit and move along edges. In fact, with the aid of graph theory, we can extend the reaction-diffusion invasion model into a compact metric graph $\mathcal {G} = (V, E)$. The graph consists of vertices $V= \{1, 2, \cdots , n\}$ and an edge set *E*. For any edge e∈E, every point ξ on the edge is described by a pair of coordinates $\xi \in (0, |e|)$. A function $f:~ \mathcal {G} \rightarrow \mathbb {R}$ is composed with a collection of functions $\{f_e|~e\in E\}$ where $f_e$ is defined by $f_e:~ (0, |e|)\rightarrow \mathbb {R}$.

For a given graph function $f:~ \mathcal {G}\rightarrow \mathbb {R}$, we define the graph integral and graph derivative as follows:

1.1 $$\begin{aligned} \int _\mathcal {G} f(x)dx:=\sum _{e\in E} \int _0^{|e|}f_e(x)dx, \;\;\; \nabla _\omega f_e(x):= \partial _x f_e(x), \end{aligned}$$

then the graph Laplacian is defined by

1.2 $$\begin{aligned} \Delta _{\omega }f_e(x):=\partial _{xx} f_e(x). \end{aligned}$$

We define the inward pointed normal derivative of *f* at the end points of the edge *e* as follows:

1.3 $$\begin{aligned} \partial _\nu f(\vartheta )= {\left\{ \begin{array}{ll} f'_e(0) & \vartheta =(e, 0), \\ - f'_e(|e|) & \vartheta =(e, |e|). \end{array}\right. } \end{aligned}$$

Denote by $U_1(x, t)$ the density of the native mosquito (*Ae. aegypti*) and $U_2(x, t)$ the density of the invasive mosquito (*Ae. albopictus*) at space location *x* and time *t*, respectively. In line with the metric-graph framework in Becker et al. (2021), we assume that $U_1(x,t)$ and $U_2(x,t)$ are subject to the natural Neumann–Kirchhoff vertex conditions. More precisely, they satisfy the following edge-wise regularity: $U_i$ is continuous on the whole graph and, for each edge *e*, ${U_i}_e$ is twice continuously differentiable in *x* and once continuously differentiable in *t* in the interior of *e*; moreover, $\partial _{x}{U_i}_e$ admits one-sided limits at the endpoints, so that the inward normal derivatives $\partial _\nu {U_i}_e(\vartheta ,t)$ defined in (1.3) are well defined. They also satisfy

1.4 $$\begin{aligned} \begin{array}{ll} \text {continuity condition: } {U_i}_e(\vartheta ,t)={U_i}_{e'}(\vartheta ,t)\quad \forall e,e'\sim \vartheta , ~i=1,2, & \vartheta \in V,\\ \text {Neumann--Kirchhoff condition: } \displaystyle \sum _{e\sim \vartheta }\partial _\nu {U_i}_e(\vartheta ,t)=0,~i=1,2, & \vartheta \in V. \end{array} \end{aligned}$$

Thus, we construct a two-species competition model in a compact metric graph:

1.5 $$\begin{aligned} {\left\{ \begin{array}{ll} \frac{\partial U_1}{\partial t}- D_1\Delta _\omega U_1=r_1U_1\left( 1-\displaystyle {\frac{U_1}{K_1}}\right) - \tilde{a}_1U_1U_2, & (x, t)\in \mathcal {G}\times (0, +\infty ), \\ \frac{\partial U_2}{\partial t}- D_2\Delta _\omega U_2= r_2U_2\left( 1-\displaystyle {\frac{U_2}{K_2}}\right) -\tilde{a}_2U_1U_2, & (x, t)\in \mathcal {G}\times (0, +\infty ), \\ {U_i}_e(\vartheta ,t)={U_i}_{e'}(\vartheta ,t)\quad \forall e,e'\sim \vartheta ,~i=1,2, & (\vartheta , t)\in V\times (0, +\infty ),\\ \displaystyle \sum _{e\sim \vartheta }\partial _\nu {U_i}_e(\vartheta ,t)= 0,~i=1,2, & (\vartheta , t)\in V\times (0, +\infty ),\\ U_1(x, 0)=U_{10}(x)\ge (\not \equiv ) 0, ~ U_2(x, 0)=U_{20}(x)\ge (\not \equiv ) 0, & x\in \mathcal {G}. \end{array}\right. } \end{aligned}$$

Here $D_1$ and $D_2$ denote the diffusion coefficients for the two species, respectively. The intrinsic growth rates are denoted by $r_1$ and $r_2$, while $K_1$ and $K_2$ represent their respective carrying capacities. The interspecific competition coefficients are given by $\tilde{a}_1$ and $\tilde{a}_2$. $\Delta _\omega$ signifies the graph Laplacian operator as defined in (1.2).

In order to minimize the number of parameters involved in the model, we introduce the dimensionless variables. Set

1.6 $$\begin{aligned} u_1=\frac{1}{K_1}U_1,~ u_2=\frac{1}{K_2}U_2, ~\bar{t}= r_1 t. \end{aligned}$$

Then omitting the bar of *t*, system (1.5) is rewritten as follows:

1.7 $$\begin{aligned} {\left\{ \begin{array}{ll} \frac{\partial u_1}{\partial t}- d_1\Delta _\omega u_1=u_1(1-u_1-a_1u_2), & (x, t)\in \mathcal {G}\times (0, +\infty ), \\ \frac{\partial u_2}{\partial t}- d_2\Delta _\omega u_2= u_2(r-a_2u_1-ru_2), & (x, t)\in \mathcal {G}\times (0, +\infty ), \\ {u_i}_e(\vartheta ,t)={u_i}_{e'}(\vartheta ,t) \quad \forall e,e'\sim \vartheta ,~i=1,2, & (\vartheta , t)\in V\times (0, +\infty ),\\ \displaystyle \sum _{e\sim \vartheta }\partial _\nu {u_i}_e(\vartheta ,t)= 0,~i=1,2, & (\vartheta , t)\in V\times (0, +\infty ),\\ u_1(x, 0)=u_{10}(x)\ge (\not \equiv ) 0, ~ u_2(x, 0)=u_{20}(x)\ge (\not \equiv ) 0, & x\in \mathcal {G}, \end{array}\right. } \end{aligned}$$

where

1.8 $$\begin{aligned} a_1=\frac{K_2\tilde{a}_1}{r_1}, r=\frac{r_2}{r_1}, a_2=\frac{K_1\tilde{a}_2}{r_1}, d_1=\frac{D_1}{r_1}, d_2=\frac{D_2}{r_1}. \end{aligned}$$

The primary objective of this paper is to investigate the invasion of *Ae. albopictus* along Route 441 in Florida. In He and Ni (2016), He and Ni analyzed system (1.7) under classical Laplacian diffusion. For scenarios of weak-strong competition ($a_1>1$ and $a_2<r$) and strong-weak competition ($a_1<1$ and $a_2>r$), solutions of the system with classical Laplacian diffusion were shown to converge globally asymptotically to the semi-positive equilibria (0, 1) and (1, 0), respectively. Under weak-weak competition ($a_1<1$ and $a_2<r$), solutions of the system with classical Laplacian diffusion converge globally asymptotically to a unique positive equilibrium. This work extends the global stability results of solutions from the classical Laplacian diffusion system to the graph Laplacian diffusion system. Additionally, numerical simulations are employed to characterize the travelling waves under some diffusion rate conditions.

Reaction–diffusion equations on metric graphs have been studied in various scenarios; for example, Lou and Morita (2024) investigated qualitative properties of solutions on noncompact graph structures. While their work addresses different geometric settings and employs different techniques, the present paper focuses on a coupled two-species competition system posed on a compact metric graph. On this compact metric graph, we establish maximum and comparison principles. We primarily use them as technical tools to study well-posedness and monotone iteration schemes for the nonlinear system arising from biological competition dynamics.

The rest of the article is structured as follows: Section 2 generalizes the maximum principle to the reaction-diffusion equations on a compact metric graph. In section 3 we prove the global existence and uniqueness of solutions to system (1.7). Section 4 deals with global stability of solutions under varying competitive intensities. Section 5 presents numerical experiments to simulate the spread of *Ae. albopictus* in Florida via State Route 441. Discussion and conclusions are given in section 6.

## Maximum principle and comparison principle

In this section, we establish the strong maximum principle and the comparison principle for scalar reaction–diffusion equations on compact metric graphs.

### Lemma 2.1

(Strong Maximum Principle) Let d>0 and $K\in \mathbb {R}$ be constants, and let T>0. Assume that $u\in C(\mathcal {G}\times [0,T])$, and for each edge *e*,

$$ u_e\in C^{2,1}(e^\circ \times (0,T])\cap C({\overline{e}}\times [0,T]). $$

Suppose that

2.1 $$\begin{aligned} {\left\{ \begin{array}{ll} \frac{\partial u}{\partial t} -d\Delta _\omega u+Ku\ge 0, & (x,t)\in \mathcal {G}\times (0,T],\\ {u}_e(\vartheta ,t)= {u}_{e'}(\vartheta ,t) \quad \forall e,e'\sim \vartheta , & (\vartheta , t)\in V\times (0, T],\\ \displaystyle \sum _{e\sim \vartheta }\partial _\nu u_e(\vartheta ,t)\ge 0, & (\vartheta , t)\in V\times (0,T],\\ u(x,0)\ge 0, & x\in \mathcal {G}. \end{array}\right. } \end{aligned}$$

Then u(x,t)≥0 for all $(x,t)\in \mathcal {G}\times (0,T]$. Moreover, if $u(\cdot ,0)\not \equiv 0$, then

$$ u(x,t)>0\qquad \text {for all }(x,t)\in \mathcal {G}\times (0,T]. $$

### Proof

Set

$$ w(x,t)=e^{-Kt}u(x,t), \qquad (x,t)\in \mathcal {G}\times [0,T]. $$

Then

$$ \partial _t w-d\Delta _\omega w =e^{-Kt}\big (\partial _tu-d\Delta _\omega u+Ku\big )\ge 0 \quad \text {in }\mathcal {G}\times (0,T]. $$

Since the factor $e^{-Kt}$ is independent of *x*, the vertex conditions are preserved:

$$ w_e(\vartheta ,t)=w_{e'}(\vartheta ,t)\quad \forall e,e'\sim \vartheta , $$

and

$$ \sum _{e\sim \vartheta }\partial _\nu w_e(\vartheta ,t) =e^{-Kt}\sum _{e\sim \vartheta }\partial _\nu u_e(\vartheta ,t)\ge 0 \qquad \text {for }(\vartheta ,t)\in V\times (0,T]. $$

Also,

$$ w(x,0)=u(x,0)\ge 0\qquad \text {on }\mathcal {G}. $$

We first prove that w≥0 on $\mathcal {G}\times [0,T]$. Assume by contradiction that

$$ m:=\min _{\mathcal {G}\times [0,T]} w<0. $$

Because $w(\cdot ,0)\ge 0$, this minimum is attained at some point $(x_0,t_0)\in \mathcal {G}\times (0,T]$. We consider two cases.

(i) $x_0$* lies in the interior of some edge.* Then $(x_0,t_0)$ is an interior minimum point of *w* in the usual parabolic sense. Hence

$$ \partial _t w(x_0,t_0)\le 0,\qquad \Delta _\omega w(x_0,t_0)\ge 0, $$

so that

$$ (\partial _t w-d\Delta _\omega w)(x_0,t_0)\le 0. $$

Since $\partial _t w-d\Delta _\omega w\ge 0$, we actually obtain

$$ (\partial _t w-d\Delta _\omega w)(x_0,t_0)=0. $$

By the classical parabolic strong maximum principle on the edge, *w* must be constant in a neighborhood of $(x_0,t_0)$ on that edge. By connectedness and the vertex continuity condition, this propagation argument implies that $w(\cdot ,t_0)\equiv m<0$ on $\mathcal {G}$, which contradicts $w(\cdot ,0)\ge 0$ together with the parabolic maximum principle in time. Therefore, this case cannot occur unless w≡m<0 on $\mathcal {G}\times [0,t_0]$, which is impossible.

(ii) $x_0=\vartheta \in V$* is a vertex.* Since $w_e(\vartheta ,t_0)=w_{e'}(\vartheta ,t_0)$ for all $e,e'\sim \vartheta$, the value $w(\vartheta ,t_0)=m$ is the common boundary minimum of the restrictions $w_e(\cdot ,t_0)$ on all incident edges. If *w* is not constant in a space-time neighborhood of $(\vartheta ,t_0)$ along some incident edge *e*, then by the parabolic boundary-point Hopf lemma applied on that edge we obtain

$$ \partial _\nu w_e(\vartheta ,t_0)<0. $$

Applying the same argument to every incident edge yields

$$ \partial _\nu w_e(\vartheta ,t_0)<0\qquad \text {for all }e\sim \vartheta . $$

Hence

$$ \sum _{e\sim \vartheta }\partial _\nu w_e(\vartheta ,t_0)<0, $$

which contradicts the vertex condition

$$ \sum _{e\sim \vartheta }\partial _\nu w_e(\vartheta ,t_0)\ge 0. $$

Therefore the vertex case is also impossible.

Combining the two cases, we conclude that

$$ w(x,t)\ge 0\qquad \text {for all }(x,t)\in \mathcal {G}\times [0,T]. $$

Hence

$$ u(x,t)=e^{Kt}w(x,t)\ge 0 \qquad \text {for all }(x,t)\in \mathcal {G}\times [0,T]. $$

It remains to prove the strict positivity when $u(\cdot ,0)\not \equiv 0$. Equivalently, assume $w(\cdot ,0)\not \equiv 0$. If there exists $(x_1,t_1)\in \mathcal {G}\times (0,T]$ such that $w(x_1,t_1)=0$, then since w≥0, the point $(x_1,t_1)$ is a global minimum point of *w*. Repeating the above argument, together with the strong maximum principle on each edge and the vertex continuity condition, we infer that $w(\cdot ,t)\equiv 0$ on $\mathcal {G}\times [0,t_1]$, which contradicts $w(\cdot ,0)\not \equiv 0$ and $w(\cdot ,0)\ge 0$. Therefore

$$ w(x,t)>0\qquad \text {for all }(x,t)\in \mathcal {G}\times (0,T]. $$

Consequently,

$$ u(x,t)=e^{Kt}w(x,t)>0 \qquad \text {for all }(x,t)\in \mathcal {G}\times (0,T]. $$

This completes the proof. □

In light of Lemma 2.1, we derive the following comparison principle.

### Lemma 2.2

(Comparison Principle) Let d>0, α>0, and β>0 be constants, and let T>0. Assume that ${\overline{u}}, {\underline{u}}\in C(\mathcal {G}\times [0,T])$, and for each edge *e*,

$$ {\overline{u}}_e,\ {\underline{u}}_e\in C^{2,1}(e^\circ \times (0,T])\cap C({\overline{e}}\times [0,T]). $$

Suppose that

2.2 $$\begin{aligned} {\left\{ \begin{array}{ll} \frac{\partial {\overline{u}}}{\partial t}-d\Delta _\omega {\overline{u}}\ge {\overline{u}}(\alpha -\beta {\overline{u}}), & (x,t)\in \mathcal {G}\times (0, T],\\ \partial _t {\underline{u}}-d\Delta _\omega {\underline{u}} \le {\underline{u}}(\alpha -\beta {\underline{u}}), & (x,t)\in \mathcal {G}\times (0, T],\\ \underline{u}_e(\vartheta ,t)= \underline{u}_{e'}(\vartheta ,t),~ \overline{u}_e(\vartheta ,t)= \overline{u}_{e'}(\vartheta ,t) \quad \forall e,e'\sim \vartheta , & (\vartheta , t)\in V\times (0, T],\\ \displaystyle \sum _{e\sim \vartheta }\partial _\nu {\underline{u}}_e(\vartheta , t)\le 0\le \sum _{e\sim \vartheta }\partial _\nu {\overline{u}}_e(\vartheta , t), & (\vartheta , t)\in V\times (0,T],\\ {\overline{u}}(x,0)\ge {\underline{u}}(x,0), & x\in \mathcal {G}. \end{array}\right. }\nonumber \\ \end{aligned}$$

Then $${\overline{u}}(x,t)\ge {\underline{u}}(x,t)$$ in $$\mathcal {G}\times [0,T]$$.

### Proof

Set $v={\overline{u}}-{\underline{u}}$. From (2.2) we obtain

$$ \frac{\partial v}{\partial t}-d\Delta _\omega v \ge {\overline{u}}(\alpha -\beta {\overline{u}})-{\underline{u}}(\alpha -\beta {\underline{u}}) =(\alpha -\beta ({\overline{u}}+{\underline{u}}))\,v \quad \text {in }\mathcal {G}\times (0,T], $$

together with

$$ \sum _{e\sim \vartheta }\partial _\nu v_e(\vartheta ,t) =\sum _{e\sim \vartheta }\partial _\nu {\overline{u}}_e(\vartheta ,t) -\sum _{e\sim \vartheta }\partial _\nu {\underline{u}}_e(\vartheta ,t)\ge 0, \qquad v(\cdot ,0)\ge 0. $$

Since $\mathcal {G}$ is finite, ${\overline{u}}$ and ${\underline{u}}$ are bounded on $\mathcal {G}\times [0,T]$, we may choose

$$ K:=\Vert \alpha -\beta ({\overline{u}}+{\underline{u}})\Vert _{L^\infty (\mathcal {G}\times (0,T])}+1, $$

so that $K+\alpha -\beta ({\overline{u}}+{\underline{u}})\ge 1$ on $\mathcal {G}\times (0,T]$. Then

$$ \frac{\partial v}{\partial t}-d\Delta _\omega v+K v\ge 0 \quad \text {in }\mathcal {G}\times (0,T]. $$

Applying Lemma 2.1 yields v≥0 in $\mathcal {G}\times [0,T]$, and the proof is complete. □

## Existence and uniqueness

For simplicity, we denote $\textbf{f}(\textbf{u})=(f_1(u_1, u_2), f_2(u_1, u_2))$ throughout this paper, here

$$\begin{aligned} f_1(u_1, u_2)=u_1(1- u_1-a_1u_2),\quad f_2(u_1, u_2)=u_2(r-a_2u_1-r u_2). \end{aligned}$$

First, we define the coupled upper and lower solutions for system (1.7).

### Definition 3.1

Assume that  for i=1,2, and for each edge *e*, $\tilde{u}_{ie}$ and , then a pair of functions $\tilde{\textbf{u}}=(\tilde{u}_1, \tilde{u}_2)$ and  are called *coupled upper and lower solutions* of (1.7) if  and3.1

Given a pair of coupled upper and lower solutions $\tilde{\textbf{u}}$ and , we define the ordered interval3.2Choose constants

$$\begin{aligned} K_1=\max _{\textbf{u}\in \Lambda }\big |1-2u_1-a_1u_2\big |,\qquad K_2=\max _{\textbf{u}\in \Lambda }\big |r-a_2u_1-2ru_2\big |. \end{aligned}$$

Then

3.3 $$\begin{aligned} K_i+\frac{\partial f_i}{\partial u_i}(\textbf{u})\ge 0 \qquad \text {for all }\textbf{u}\in \Lambda ,\ i=1,2. \end{aligned}$$

For i=1,2, set

3.4 $$\begin{aligned} F_i(u_1,u_2)=K_i u_i+f_i(u_1,u_2). \end{aligned}$$

Consider the shifted system

3.5 $$\begin{aligned} {\left\{ \begin{array}{ll} \frac{\partial u_1}{\partial t}- d_1\Delta _\omega u_1+K_1 u_1=F_1(u_1,u_2), & (x,t)\in \mathcal {G}\times (0, T],\\ \frac{\partial u_2}{\partial t}- d_2\Delta _\omega u_2+K_2 u_2=F_2(u_1,u_2), & (x,t)\in \mathcal {G}\times (0, T],\\ {u_i}_e(\vartheta ,t)={u_i}_{e'}(\vartheta ,t) \quad \forall e,e'\sim \vartheta ,\ i=1,2, & (\vartheta , t)\in V\times (0, T],\\ \displaystyle \sum _{e\sim \vartheta }\partial _\nu (u_i)_e(\vartheta ,t)=0,\ i=1,2, & (\vartheta , t)\in V\times (0,T],\\ u_1(x,0)=u_{10}(x)\ge 0,\quad u_2(x,0)=u_{20}(x)\ge 0, & x\in \mathcal {G}. \end{array}\right. }\nonumber \\ \end{aligned}$$

Clearly, (3.5) is equivalent to (1.7) on any finite time interval.

We now construct monotone sequences between  and $\tilde{\textbf{u}}$. Let  and $\overline{\textbf{u}}^{(0)}=\tilde{\textbf{u}}$. For m≥1, define $\overline{\textbf{u}}^{(m)}=({\overline{u}}_1^{(m)},{\overline{u}}_2^{(m)})$ and $\underline{\textbf{u}}^{(m)}=({\underline{u}}_1^{(m)},{\underline{u}}_2^{(m)})$ as the unique solutions of

3.6 $$\begin{aligned} {\left\{ \begin{array}{ll} \frac{\partial {\overline{u}}_1^{(m)}}{\partial t}- d_1\Delta _\omega {\overline{u}}_1^{(m)}+K_1 {\overline{u}}_1^{(m)} =F_1({\overline{u}}_1^{(m-1)}, {\underline{u}}_2^{(m-1)}), & (x,t)\in \mathcal {G}\times (0, T],\\ \frac{\partial {\overline{u}}_2^{(m)}}{\partial t}- d_2\Delta _\omega {\overline{u}}_2^{(m)}+K_2 {\overline{u}}_2^{(m)} =F_2({\underline{u}}_1^{(m-1)}, {\overline{u}}_2^{(m-1)}), & (x,t)\in \mathcal {G}\times (0, T],\\ \frac{\partial {\underline{u}}_1^{(m)}}{\partial t}- d_1\Delta _\omega {\underline{u}}_1^{(m)}+K_1 {\underline{u}}_1^{(m)} =F_1({\underline{u}}_1^{(m-1)}, {\overline{u}}_2^{(m-1)}), & (x,t)\in \mathcal {G}\times (0, T],\\ \frac{\partial {\underline{u}}_2^{(m)}}{\partial t}- d_2\Delta _\omega {\underline{u}}_2^{(m)}+K_2 {\underline{u}}_2^{(m)} =F_2({\overline{u}}_1^{(m-1)}, {\underline{u}}_2^{(m-1)}), & (x,t)\in \mathcal {G}\times (0, T],\\ {{\overline{u}}_{ie}^{(m)}}(\vartheta ,t)={{\overline{u}}_{ie'}^{(m)}}(\vartheta ,t),~ {{\underline{u}}_{ie}^{(m)}}(\vartheta ,t)={{\underline{u}}_{ie'}^{(m)}}(\vartheta ,t) \quad \forall e,e'\sim \vartheta ,\ i=1,2, & (\vartheta , t)\in V\times (0, T],\\ \displaystyle \sum _{e\sim \vartheta }\partial _\nu ({\overline{u}}_i^{(m)})_e(\vartheta ,t)=0,\quad \sum _{e\sim \vartheta }\partial _\nu ({\underline{u}}_i^{(m)})_e(\vartheta ,t)=0,\ i=1,2, & (\vartheta , t)\in V\times (0,T],\\ {\overline{u}}_i^{(m)}(x,0)={\underline{u}}_i^{(m)}(x,0)=u_{i0}(x),\ i=1,2, & x\in \mathcal {G}. \end{array}\right. }\nonumber \\ \end{aligned}$$

Existence and uniqueness of $\overline{\textbf{u}}^{(m)}$ and $\underline{\textbf{u}}^{(m)}$ follow from the linear parabolic theory on compact metric graphs, see Becker et al. (2021).

Moreover, by (Becker et al. (2021), Theorem 4), each component admits a Duhamel formula in terms of the heat kernel $H_i(t,x,y)$ associated with $d_i\Delta _\omega$ under the Neumann–Kirchhoff vertex conditions, for example,

3.7 $$\begin{aligned} {\overline{u}}_1^{(m)}(x,t)&=e^{-K_1 t}\int _{\mathcal {G}} H_1(t,x,y)\,u_{10}(y)\,dy \nonumber \\&\quad +\int _0^t e^{-K_1(t-\tau )}\int _{\mathcal {G}} H_1(t-\tau ,x,y)\, F_1({\overline{u}}_1^{(m-1)},{\underline{u}}_2^{(m-1)})(y,\tau )\,dy\,d\tau , \end{aligned}$$

and analogous representations hold for ${\underline{u}}_1^{(m)},{\overline{u}}_2^{(m)}$ and ${\underline{u}}_2^{(m)}$. In particular, the iterates are well-defined on $\mathcal {G}\times [0,T]$.

Since the nonlinearities are mixed monotone on the ordered interval Λ and the vertex conditions in Definition 3.1 are compatible with Lemma 2.2, a standard monotone-iteration argument (cf. Smith (2008)) yields the following lemma.

### Lemma 3.1

The sequences $\{\overline{\textbf{u}}^{(m)}\}_{m\ge 1}$ and $\{\underline{\textbf{u}}^{(m)}\}_{m\ge 1}$ generated by (3.6) satisfy that, for all $(x,t)\in \mathcal {G}\times [0,T]$,3.8Moreover, for each m≥1, $\overline{\textbf{u}}^{(m)}$ and $\underline{\textbf{u}}^{(m)}$ are coupled upper and lower solutions of (1.7).

In view of Lemma 3.1, the pointwise limits

3.9 $$\begin{aligned} \lim _{m\rightarrow \infty }\overline{\textbf{u}}^{(m)}=\overline{\textbf{u}},\qquad \lim _{m\rightarrow \infty }\underline{\textbf{u}}^{(m)}=\underline{\textbf{u}} \end{aligned}$$

exist on $\mathcal {G}\times [0,T]$. In the following theorem, we demonstrate that $({\overline{u}}_1,{\underline{u}}_2)$ and $({\underline{u}}_1,{\overline{u}}_2)$ are in fact solutions of (1.7).

### Theorem 3.1

Let $\tilde{\textbf{u}}$ and  be a pair of coupled upper and lower solutions of (1.7), bounded on $\mathcal {G}\times [0,T]$. Let $\{\overline{\textbf{u}}^{(m)}\}_{m\ge 1}$ and $\{\underline{\textbf{u}}^{(m)}\}_{m\ge 1}$ be generated by (3.6), and let $\overline{\textbf{u}},\underline{\textbf{u}}$ be defined by (3.9). Then $({\overline{u}}_1,{\underline{u}}_2)$ and $({\underline{u}}_1,{\overline{u}}_2)$ are solutions of (1.7) on [0, *T*]. Moreover, $\overline{\textbf{u}}=\underline{\textbf{u}}$ on $\mathcal {G}\times [0,T]$. In particular, (1.7) admits a unique solution between  and $\tilde{\textbf{u}}$.

### Proof

By Lemma 3.1, the sequences $\{\overline{\textbf{u}}^{(m)}\}$ and $\{\underline{\textbf{u}}^{(m)}\}$ are monotone and uniformly bounded between  and $\tilde{\textbf{u}}$ on $\mathcal {G}\times [0,T]$. Hence the pointwise limits $\overline{\textbf{u}}$ and $\underline{\textbf{u}}$ in (3.9) exist.

For each *m*, ${\overline{u}}_i^{(m)}$ solves the linear problem in (3.6) under the Neumann–Kirchhoff vertex conditions. Let $H_i(t,x,y)$ be the heat kernel associated with $d_i\Delta _\omega$ on $\mathcal {G}$. By (Becker et al. (2021), Theorem 4), ${\overline{u}}_i^{(m)}$ admits the Duhamel formula

3.10 $$\begin{aligned} {\overline{u}}_i^{(m)}(x,t)&=e^{-K_i t}\int _{\mathcal {G}}H_i(t,x,y)\,u_{i0}(y)\,dy\nonumber \\&\quad +\int _0^t e^{-K_i(t-\tau )}\int _{\mathcal {G}}H_i(t-\tau ,x,y)\, F_i(\Pi _i^{(m-1)})(y,\tau )\,dy\,d\tau , \end{aligned}$$

where $\Pi _1^{(m-1)}=({\overline{u}}_1^{(m-1)},{\underline{u}}_2^{(m-1)})$ and $\Pi _2^{(m-1)}=({\underline{u}}_1^{(m-1)},{\overline{u}}_2^{(m-1)})$. An analogous representation holds for ${\underline{u}}_i^{(m)}$. Since $H_i\ge 0$, $\int _{\mathcal {G}}H_i(t,x,y)\,dy=1$, and all iterates are uniformly bounded on $\mathcal {G}\times [0,T]$, we may pass to the limit in (3.10) by dominated convergence to obtain, for $(x,t)\in \mathcal {G}\times (0,T]$, that

$$\begin{aligned} {\overline{u}}_1(x,t)&=e^{-K_1 t}\int _{\mathcal {G}}H_1(t,x,y)\,u_{10}(y)\,dy\\&\quad +\int _0^t e^{-K_1(t-\tau )}\int _{\mathcal {G}}H_1(t-\tau ,x,y)\, F_1({\overline{u}}_1,{\underline{u}}_2)(y,\tau )\,dy\,d\tau , \\ {\underline{u}}_2(x,t)&=e^{-K_2 t}\int _{\mathcal {G}}H_2(t,x,y)\,u_{20}(y)\,dy\\&\quad +\int _0^t e^{-K_2(t-\tau )}\int _{\mathcal {G}}H_2(t-\tau ,x,y)\, F_2({\overline{u}}_1,{\underline{u}}_2)(y,\tau )\,dy\,d\tau , \end{aligned}$$

and similarly for $({\underline{u}}_1,{\overline{u}}_2)$. Therefore $({\overline{u}}_1,{\underline{u}}_2)$ and $({\underline{u}}_1,{\overline{u}}_2)$ satisfy (3.5), hence solve (1.7) on $\mathcal {G}\times (0,T]$ with the Neumann–Kirchhoff vertex conditions.

Next, we set

$$ p(x,t)={\overline{u}}_1(x,t)-{\underline{u}}_1(x,t),\quad q(x,t)={\overline{u}}_2(x,t)-{\underline{u}}_2(x,t), \quad W(t)=\Vert p(\cdot ,t)\Vert _{L^\infty (\mathcal {G})}+\Vert q(\cdot ,t)\Vert _{L^\infty (\mathcal {G})}. $$

Since $\overline{\textbf{u}}$ and $\underline{\textbf{u}}$ take values in the ordered interval Λ, there exist constants $K_3,K_4>0$ (depending only on Λ) such that for all $(x,t)\in \mathcal {G}\times [0,T]$,

3.11 $$\begin{aligned} F_1({\overline{u}}_1,{\underline{u}}_2)-F_1({\underline{u}}_1,{\overline{u}}_2)&\le K_1\,p + K_3\,q, \end{aligned}$$

3.12 $$\begin{aligned} F_2({\underline{u}}_1,{\overline{u}}_2)-F_2({\overline{u}}_1,{\underline{u}}_2)&\le K_4\,p + K_2\,q. \end{aligned}$$

Substituting the Duhamel formulas for ${\overline{u}}_1$ and ${\underline{u}}_1$ yields

3.13 $$\begin{aligned} p(x,t)&\le \int _0^t e^{-K_1(t-\tau )}\int _{\mathcal {G}}H_1(t-\tau ,x,y)\, \big (K_1 p(y,\tau )+K_3 q(y,\tau )\big )\,dy\,d\tau . \end{aligned}$$

Using $\int _{\mathcal {G}}H_1(\cdot )\,dy=1$, we get

3.14 $$\begin{aligned} \Vert p(\cdot ,t)\Vert _{L^\infty (\mathcal {G})} \le \frac{1-e^{-K_1 t}}{K_1}\Big (K_1\Vert p(\cdot ,t)\Vert _{L^\infty (\mathcal {G})}+K_3\Vert q(\cdot ,t)\Vert _{L^\infty (\mathcal {G})}\Big ). \end{aligned}$$

Similarly,

3.15 $$\begin{aligned} \Vert q(\cdot ,t)\Vert _{L^\infty (\mathcal {G})} \le \frac{1-e^{-K_2 t}}{K_2}\Big (K_4\Vert p(\cdot ,t)\Vert _{L^\infty (\mathcal {G})}+K_2\Vert q(\cdot ,t)\Vert _{L^\infty (\mathcal {G})}\Big ). \end{aligned}$$

Adding (3.14) and (3.15) yields

3.16 $$\begin{aligned} W(t)\le \theta (t)\,W(t), \qquad \theta (t):=\max \left\{ \frac{1-e^{-K_1 t}}{K_1}\max \left\{ K_1,K_3\right\} , \frac{1-e^{-K_2 t}}{K_2}\max \left\{ K_2,K_4\right\} \right\} . \end{aligned}$$

Choose $T_1\in (0,T]$ such that $\theta (T_1)\le \frac{1}{2}$. Then (3.16) implies W(t)=0 for all $t\in [0,T_1]$, i.e.

$$ {\overline{u}}_1={\underline{u}}_1,\qquad {\overline{u}}_2={\underline{u}}_2 \quad \text {on }\mathcal {G}\times [0,T_1]. $$

Hence $({\overline{u}}_1,{\underline{u}}_2)\equiv ({\underline{u}}_1,{\overline{u}}_2)$ is the unique solution of (1.7) on $[0,T_1]$ between  and $\tilde{\textbf{u}}$.

The constant $T_1$ depends only on the bounds of Λ (hence only on  and $\tilde{\textbf{u}}$ on [0, *T*]), and not on the initial functions. Therefore we may repeat the same argument at time $T_1$ with initial value $\textbf{u}(\cdot ,T_1)$ and obtain the uniqueness on $[T_1,2T_1]$. Iterating finitely many times covers the whole interval [0, *T*]. Consequently, $\overline{\textbf{u}}=\underline{\textbf{u}}$ on $\mathcal {G}\times [0,T]$, and the solution of (1.7) is unique. □

We extend the local solutions derived in Theorem 3.1 to its maximal existence time. To accomplish this, the following *a priori* estimates are required.

### Lemma 3.2

Let $(u_1, u_2)$ be a solution to system (1.7) defined for t∈[0,T] for some $T\in (0, +\infty )$. Then there exist constants $M_1$ and $M_2$, independent of *T*, such that

3.17 $$\begin{aligned} \begin{aligned} &  0\le u_1(x, t)\le M_1,~ (x, t)\in \mathcal {G}\times [0, T], \\  &  0\le u_2(x, t)\le M_2,~ (x, t)\in \mathcal {G}\times [0, T].\end{aligned}\end{aligned}$$

### Proof

Given the initial conditions $u_{i0}(x)\ge 0$ for i=1,2, we apply the comparison principle (Lemma 2.2) to derive

3.18 $$\begin{aligned} u_i(x, t)\ge 0 \text { for } (x, t)\in \mathcal {G}\times [0, T]. \end{aligned}$$

Consequently, since $(u_1, u_2)$ satisfies

$$\begin{aligned} {\left\{ \begin{array}{ll} \frac{\partial u_1}{\partial t}- d_1\Delta _\omega u_1=u_1(1-u_1-a_1u_2)\le u_1(1-u_1), & (x, t)\in \mathcal {G}\times (0, T], \\ \frac{\partial u_2}{\partial t}- d_2\Delta _\omega u_2=u_2(r-a_2u_1-ru_2)\le u_2(r-ru_2), & (x, t)\in \mathcal {G}\times (0, T], \\ {u_i}_e(\vartheta ,t)={u_i}_{e'}(\vartheta ,t) \quad \forall e,e'\sim \vartheta ,~i=1,2, & (\vartheta , t)\in V\times (0, T],\\ \displaystyle \sum _{e\sim \vartheta }\partial _\nu {u_i}_e(\vartheta ,t)= 0, ~i=1,2, & (\vartheta , t)\in V\times (0,T],\\ u_1(x, 0)=u_{10}(x)\ge 0,~u_2(x, 0)=u_{20}(x)\ge 0, & x\in \mathcal {G}, \end{array}\right. } \end{aligned}$$

by choosing

3.19 $$\begin{aligned} M_1=\max \left\{ \max _{x\in \mathcal {G}} u_{10}(x),~ 1\right\} \text { and } M_2=\max \left\{ \max _{x\in \mathcal {G}} u_{20}(x),~ 1\right\} , \end{aligned}$$

we know that $(M_1, M_2)$ and (0, 0) form a pair of upper and lower solutions to system (1.7). Applying Theorem 3.1 directly yields (3.17). □

By means of the *a priori* estimates established in Lemma 3.2, we now present the following global existence theorem.

### Theorem 3.2

If the initial functions satisfy

$$ u_{10},\,u_{20}\in C(\mathcal {G}), \quad u_{10}(x)\ge 0,\; u_{20}(x)\ge 0, \text { and } (u_{10},u_{20})\not \equiv (0,0), $$

then system (1.7) admits a unique solution for every $t\in [0,+\infty )$.

## Stability of solutions

The primary objective of this section is to establish the global asymptotical stability of solutions to system (1.7). Depending on the intensity of competitive interactions, we will analyze three types of competitive relationships: weak-strong competition, strong-weak competition, and weak-weak competition.

### Weak-strong competition and strong-weak competition

In this subsection, we first examine the case that $u_1$ (*Ae. aegypti*) is an inferior competitor and $u_2$ (*Ae. albopictus*) is a superior competitor; namely,

4.1 $$\begin{aligned} a_1>1 \text{ and } a_2<r. \end{aligned}$$

To demonstrate the global stability of solutions to system (1.7), we present the following lemma.

#### Lemma 4.1

Let

4.2 $$\begin{aligned} d>0, ~ \alpha>0,~ \beta >0 \end{aligned}$$

be constants. Assume that $w\in C(\mathcal {G}\times [0,T])$, and for each edge *e*,

$$ w_e\in C^{2,1}(e^\circ \times (0,+\infty ))\cap C({\overline{e}}\times [0,+\infty )). $$

If *w* satisfies

4.3 $$\begin{aligned} {\left\{ \begin{array}{ll} \frac{\partial w}{\partial t}-d\Delta _\omega w\ge ~(\le )~ w(\alpha -\beta w), & (x, t)\in \mathcal {G}\times (0, +\infty ), \\ {w}_e(\vartheta ,t)={w}_{e'}(\vartheta ,t) \quad \forall e,e'\sim \vartheta , & (\vartheta , t)\in V\times (0, +\infty ),\\ \displaystyle \sum _{e\sim \vartheta }\partial _\nu w_e(\vartheta ,t)\ge ~(\le ) 0, & (\vartheta , t)\in V\times (0, +\infty ),\\ w(x, 0)=w_{0}(x)\ge (\not \equiv )0, & x\in \mathcal {G}, \end{array}\right. } \end{aligned}$$

then for any given ε>0 there exists $t_\varepsilon >0$ such that

4.4 $$\begin{aligned} w(x,t)>\frac{\alpha }{\beta }-\varepsilon ~(w(x,t)<\frac{\alpha }{\beta }+\varepsilon ), ~(x,t)\in \mathcal {G}\times [t_\varepsilon , +\infty ). \end{aligned}$$

Moreover,

4.5 $$\begin{aligned} &  \liminf _{t\rightarrow +\infty } w(x,t)\ge \frac{\alpha }{\beta } ~ (\limsup _{t\rightarrow +\infty } w(x,t)\le \frac{\alpha }{\beta }) \text{ uniformly } \text{ for } x\in \mathcal {G}. \end{aligned}$$

#### Proof

We first prove that solutions to the following scalar equation

4.6 $$\begin{aligned} {\left\{ \begin{array}{ll} \frac{\partial z}{\partial t}-d\Delta _\omega z = z(\alpha -\beta z), & (x, t)\in \mathcal {G}\times (0, +\infty ), \\ z_e(\vartheta ,t)={z}_{e'}(\vartheta ,t) \quad \forall e,e'\sim \vartheta , & (\vartheta , t)\in V\times (0, +\infty ),\\ \displaystyle \sum _{e\sim \vartheta }\partial _\nu z_e(\vartheta ,t)= 0, & (\vartheta ,t)\in V\times (0, +\infty ),\\ z(x, 0)=w_{0}(x)\ge 0, & x\in \mathcal {G}, \end{array}\right. } \end{aligned}$$

converge to $\frac{\alpha }{\beta }$ uniformly for $x\in \mathcal {G}$.

Since $w_{0}(x)\not \equiv 0$ for $x\in \mathcal {G}$, the strong maximum principle (Lemma 2.1) implies that z(x,t)>0 for $(x, t)\in \mathcal {G}\times (0, +\infty )$. For any small $t_1>0$, we set $\delta =\min _{x\in \mathcal {G}} z(x, t_1)$, then δ>0. Consider ${\underline{z}}(x, t)$ satisfying the following equation:

4.7 $$\begin{aligned} {\left\{ \begin{array}{ll} \frac{\partial {\underline{z}}}{\partial t}-d\Delta _\omega {\underline{z}}={\underline{z}}(\alpha -\beta {\underline{z}}), & (x, t)\in \mathcal {G}\times (t_1, +\infty ), \\ {\underline{z}}_e(\vartheta ,t)=\underline{z}_{e'}(\vartheta ,t) \quad \forall e,e'\sim \vartheta , & (\vartheta , t)\in V\times (t_1, +\infty ),\\ \displaystyle \sum _{e\sim \vartheta }\partial _\nu {\underline{z}}_e(\vartheta ,t)= 0, & (\vartheta ,t)\in V\times (t_1, +\infty ),\\ {\underline{z}}(x, t_1)=\delta , & x\in \mathcal {G}. \end{array}\right. } \end{aligned}$$

In fact, a solution of the ordinary differential equation corresponding to (4.7) converges to $\frac{\alpha }{\beta }$. Owing to the uniqueness of the solution, the solution of the ordinary differential equation is also the solution of (4.7). Hence we have

4.8 $$\begin{aligned} \lim _{t\rightarrow +\infty }{\underline{z}}(x, t)=\frac{\alpha }{\beta } \text{ uniformly } \text{ for } x\in \mathcal {G}. \end{aligned}$$

Hence ${\underline{z}}$ is a lower solution of system (4.6) with $t\in [t_1, +\infty )$. The comparison principle (Lemma 2.2) implies that $z(x, t)\ge {\underline{z}}(x, t)$ for $(x, t)\in \mathcal {G}\times [t_1, +\infty )$. Combining with (4.8), we obtain

4.9 $$\begin{aligned} &  \liminf _{t\rightarrow +\infty } z(x,t)\ge \frac{\alpha }{\beta } \text{ uniformly } \text{ for } x\in \mathcal {G}. \end{aligned}$$

On the other hand, consider ${\overline{z}}(x, t)$ satisfying the following equation:

4.10 $$\begin{aligned} {\left\{ \begin{array}{ll} \frac{d \overline{z}}{d t} =\overline{z}(\alpha -\beta \overline{z}), & (x, t)\in \mathcal {G}\times (0, +\infty ), \\ {\overline{z}}_e(\vartheta ,t)=\overline{z}_{e'}(\vartheta ,t) \quad \forall e,e'\sim \vartheta , & (\vartheta , t)\in V\times (0, +\infty ),\\ \displaystyle \sum _{e\sim \vartheta }\partial _\nu {\overline{z}}_e(\vartheta ,t)= 0, & (\vartheta ,t)\in V\times (0, +\infty ),\\ {\overline{z}}(x, t_1)=\max _{x\in \mathcal {G}} w_0(x), & x\in \mathcal {G}. \end{array}\right. } \end{aligned}$$

In fact, a solution of the ordinary differential equation corresponding to (4.10) converges to $\frac{\alpha }{\beta }$. The uniqueness of the solution implies that the solution of the ordinary differential equation is also the solution of (4.10). Thus we have

4.11 $$\begin{aligned} \lim _{t\rightarrow +\infty }{\overline{z}}(x, t)=\frac{\alpha }{\beta } \text{ uniformly } \text{ for } x\in \mathcal {G}. \end{aligned}$$

Moreover, since ${\overline{z}}$ is an upper solution of system (4.6) with $t\in [0, +\infty )$, we have $z(x, t)\le {\overline{z}}(x, t)$ for $(x, t)\in \mathcal {G}\times [0, +\infty )$. Combining with (4.11), we obtain

4.12 $$\begin{aligned} &  \limsup _{t\rightarrow +\infty } z(x,t)\le \frac{\alpha }{\beta } \text{ uniformly } \text{ for } x\in \mathcal {G}. \end{aligned}$$

Combining (4.9) and (4.12), we deduce that

4.13 $$\begin{aligned} &  \lim _{t\rightarrow +\infty } z(x,t)= \frac{\alpha }{\beta } \text{ uniformly } \text{ for } x\in \mathcal {G}. \end{aligned}$$

Next since *w* satisfies (4.3), the comparison principle (Lemma 2.2) implies (4.4), which immediately implies that (4.5) holds. This completes the proof. □

Applying a similar argument, we obtain the following lemma.

#### Lemma 4.2

Let

4.14 $$\begin{aligned} d>0, ~ \alpha>0,~ \beta >0 \end{aligned}$$

be constants. Assume that $w\in C(\mathcal {G}\times [0,T])$, and for each edge *e*,

$$ w_e\in C^{2,1}(e^\circ \times (0,+\infty ))\cap C({\overline{e}}\times [0,+\infty )). $$

If *w* satisfies

4.15 $$\begin{aligned} {\left\{ \begin{array}{ll} \frac{\partial w}{\partial t}-d\Delta _\omega w\le ~ w(\alpha -\beta w), & (x, t)\in \mathcal {G}\times (0, +\infty ), \\ w_e(\vartheta ,t)= {w}_{e'}(\vartheta ,t) \quad \forall e,e'\sim \vartheta , & (\vartheta , t)\in V\times (0, +\infty ),\\ \displaystyle \sum _{e\sim \vartheta }\partial _\nu w_e(\vartheta ,t)\le 0, & (\vartheta , t)\in V\times (0, +\infty ),\\ w(x, 0)=w_{0}(x)\ge ~0, & x\in \mathcal {G}, \end{array}\right. } \end{aligned}$$

then

4.16 $$\begin{aligned} &  \liminf _{t\rightarrow \infty } w(x,t)\le 0 \text{ uniformly } \text{ for } x\in \mathcal {G}. \end{aligned}$$

Now we state and prove our first main result in this section.

#### Theorem 4.1

(Weak-strong competition) Assume that (4.1) holds, then the solution $(u_1, u_2)$ to system (1.7) satisfies

4.17 $$\begin{aligned} \lim _{t\rightarrow \infty }(u_1, u_2)=(0, 1) \text{ uniformly } \text{ for } x\in \mathcal {G}. \end{aligned}$$

#### Proof

By Lemma 3.2, we have $0\le u_2(x, t)\le M_2$ for $(x, t)\in \mathcal {G}\times [0, +\infty )$. Then we find that $u_1$ satisfies

$$\begin{aligned} {\left\{ \begin{array}{ll} \frac{\partial u_1}{\partial t}-d_1\Delta _\omega u_1 \le u_1(1-u_1), & (x, t)\in \mathcal {G}\times (0, +\infty ), \\ u_{1e}(\vartheta ,t)=u_{1e'}(\vartheta ,t), \quad \forall e,e'\sim \vartheta , & (\vartheta , t)\in V\times (0, +\infty ),\\ \displaystyle \sum _{e\sim \vartheta }\partial _\nu {u_1}_e(\vartheta ,t)= 0, & (\vartheta , t)\in V\times (0, +\infty ),\\ u_1(x, 0)=u_{10}(x)\not \equiv 0, & x\in \mathcal {G}. \end{array}\right. } \end{aligned}$$

Applying Lemma 4.1, we have

$$\begin{aligned} \limsup _{t\rightarrow \infty } u_1(x,t)\le 1 \text{ uniformly } \text{ for } x\in \mathcal {G}. \end{aligned}$$

Consequently, for any $0<\varepsilon _1<<1$, there exists $t_1>0$ such that

4.18 $$\begin{aligned} u_1(x, t)< 1+\varepsilon _1,~ (x, t) \in \mathcal {G}\times [t_1, +\infty ). \end{aligned}$$

By (4.1), we can choose $\varepsilon _1=\frac{r-a_2}{2a_2}>0$. Plugging (4.18) into system (1.7), we know that $u_2$ satisfies

$$\begin{aligned} {\left\{ \begin{array}{ll} \frac{\partial u_2}{\partial t}-d_2\Delta _\omega u_2 \ge u_2(\frac{r-a_2}{2}-ru_2), & (x, t)\in \mathcal {G}\times (t_1, +\infty ), \\ u_{2e}(\vartheta ,t)=u_{2e'}(\vartheta ,t), \quad \forall e,e'\sim \vartheta , & (\vartheta , t)\in V\times (t_1, +\infty ),\\ \displaystyle \sum _{e\sim \vartheta }\partial _\nu {u_2}_e(\vartheta ,t)= 0, & (\vartheta , t)\in V\times (t_1, +\infty ),\\ u_2(x, t)|_{t=t_1}=u_2(x, t_1), & x\in \mathcal {G}. \end{array}\right. } \end{aligned}$$

Using Lemma 4.1, we have

$$\begin{aligned} \liminf _{t\rightarrow \infty } u_2(x,t)\ge \frac{r-a_2}{2r}:={\underline{s}}_1 \text{ uniformly } \text{ for } x\in \mathcal {G}. \end{aligned}$$

Consequently, for any $0<\varepsilon _2<<1$, there exists $t_2>t_1$ such that

4.19 $$\begin{aligned} u_2(x, t)> {\underline{s}}_1-\varepsilon _2, ~(x, t) \in \mathcal {G}\times [t_2, +\infty ). \end{aligned}$$

(i) Substituting (4.19) into system (1.7), we see that $u_1$ satisfies

4.20 $$\begin{aligned} {\left\{ \begin{array}{ll} \frac{\partial u_1}{\partial t}-d_1\Delta _\omega u_1 \le u_1(1-u_1-a_1{\underline{s}}_1+a_1\varepsilon _2), & (x, t)\in \mathcal {G}\times (t_2, +\infty ), \\ u_{1e}(\vartheta ,t)=u_{1e'}(\vartheta ,t), \quad \forall e,e'\sim \vartheta , & (\vartheta , t)\in V\times (t_2, +\infty ),\\ \displaystyle \sum _{e\sim \vartheta }\partial _\nu {u_1}_e(\vartheta ,t)= 0, & (\vartheta , t)\in V\times (t_2, +\infty ),\\ u_1(x, t)|_{t=t_2}=u_1(x, t_2), & x\in \mathcal {G}. \end{array}\right. }\nonumber \\ \end{aligned}$$

We claim that when $1-a_1{\underline{s}}_1<0$ the conclusion (4.17) holds. In this case, as long as we take $\varepsilon _2$ sufficiently small such that $1-a_1{\underline{s}}_1+a_1\varepsilon _2\le 0$, Lemma 4.2 implies that $\lim _{t\rightarrow \infty } u_1(x, t)=0$ uniformly for $x\in \mathcal {G}$. For any 0<ε<<1, there exists $t_*>t_2$ such that $0\le u_1(x,t)<\varepsilon$ in $x\in \mathcal {G}$. Hence $u_2$ satisfies

$$\begin{aligned} {\left\{ \begin{array}{ll} u_2(r-a_2\varepsilon -ru_2)\le \frac{\partial u_2}{\partial t}-d_2\Delta _\omega u_2\le u_2(r-ru_2), & (x,t)\in \mathcal {G}\times (t_*, +\infty ),\\ u_{2e}(\vartheta ,t)=u_{2e'}(\vartheta ,t), \quad \forall e,e'\sim \vartheta , & (\vartheta , t)\in V\times (t_*, +\infty ),\\ \displaystyle \sum _{e\sim \vartheta }\partial _\nu {u_2}_e(\vartheta ,t)= 0, & (\vartheta , t)\in V\times (t_*, +\infty ),\\ u_2(x, t)|_{t=t_*}=u_2(x, t_*), & x\in \mathcal {G}. \end{array}\right. } \end{aligned}$$

Applying Lemma 4.1, we have

$$\begin{aligned} 1-\frac{a_2\varepsilon }{r}\le \liminf _{t\rightarrow \infty } u_2(x,t)\le \limsup _{t\rightarrow \infty } u_2(x,t)\le 1 \text{ uniformly } \text{ for } x\in \mathcal {G}. \end{aligned}$$

Owing to the arbitrariness of ε, (4.17) is true. Now we have $1-a_1{\underline{s}}_1\ge 0$. This allows us to apply Lemma 4.1 to (4.20), for any $0<\varepsilon _3<<1$, we have

$$\begin{aligned} \limsup _{t\rightarrow \infty } u_1(x,t)< 1-a_1{\underline{s}}_1+a_1\varepsilon _2+\varepsilon _3 \text{ uniformly } \text{ for } x\in \mathcal {G}. \end{aligned}$$

By the arbitrariness of $\varepsilon _2$ and $\varepsilon _3$, it immediately follows that

$$\begin{aligned} \limsup _{t\rightarrow \infty } u_1(x,t)\le 1-a_1{\underline{s}}_1:={\overline{s}}_1 \text{ uniformly } \text{ for } x\in \mathcal {G}. \end{aligned}$$

Consequently, for any $0<\varepsilon _3<<1$, there exists $t_3>t_2$ such that

4.21 $$\begin{aligned} u_1(x, t)< {\overline{s}}_1+\varepsilon _3,~ (x, t) \in \mathcal {G}\times [t_3, +\infty ). \end{aligned}$$

(ii) Plugging (4.21) into system (1.7), we see that $u_2$ satisfies

$$\begin{aligned} {\left\{ \begin{array}{ll} \frac{\partial u_2}{\partial t}-d_2\Delta _\omega u_2 \ge u_2(r-a_2{\overline{s}}_1-a_2\varepsilon _3-ru_2), & (x, t)\in \mathcal {G}\times (t_3, \infty ), \\ u_{2e}(\vartheta ,t)=u_{2e'}(\vartheta ,t), \quad \forall e,e'\sim \vartheta , & (\vartheta , t)\in V\times (t_3, +\infty ),\\ \displaystyle \sum _{e\sim \vartheta }\partial _\nu {u_2}_e(\vartheta ,t)= 0, & (\vartheta , t)\in V\times (t_3, +\infty ),\\ u_2(x, t)|_{t=t_3}=u_2(x, t_3), & x\in \mathcal {G}. \end{array}\right. } \end{aligned}$$

Using Lemma 4.1, for any $0<\varepsilon _4<<1$, we have

$$\begin{aligned} \liminf _{t\rightarrow \infty } u_2(x,t)> 1-\frac{a_2{\overline{s}}_1}{r}-\frac{a_2\varepsilon _3}{r}-\varepsilon _4 \text{ uniformly } \text{ for } x\in \mathcal {G}. \end{aligned}$$

The arbitrariness of $\varepsilon _3$ and $\varepsilon _4$ imply that

4.22 $$\begin{aligned} \liminf _{t\rightarrow \infty } u_2(x,t)\ge 1-\frac{a_2{\overline{s}}_1}{r}:={\underline{s}}_2 \text{ uniformly } \text{ for } x\in \mathcal {G}. \end{aligned}$$

Thus, for any $0<\varepsilon _4<<1$, there exists $t_4>t_3$ such that

4.23 $$\begin{aligned} u_2(x, t)>{\underline{s}}_2-\varepsilon _4,~ (x, t) \in \mathcal {G}\times [t_4, +\infty ). \end{aligned}$$

(iii) Substituting (4.23) into system (1.7), we obtain that $u_1$ satisfies

$$\begin{aligned} {\left\{ \begin{array}{ll} \frac{\partial u_1}{\partial t}-d_1\Delta _\omega u_1 \le u_1(1-u_1-a_1{\underline{s}}_2+a_1\varepsilon _4), & (x, t)\in \mathcal {G}\times (t_4, \infty ), \\ u_{1e}(\vartheta ,t)=u_{1e'}(\vartheta ,t), \quad \forall e,e'\sim \vartheta , & (\vartheta , t)\in V\times (t_4, +\infty ),\\ \displaystyle \sum _{e\sim \vartheta }\partial _\nu {u_1}_e(\vartheta ,t)= 0, & (\vartheta , t)\in V\times (t_4, +\infty ),\\ u_1(x, t)|_{t=t_4}=u_1(x, t_4), & x\in \mathcal {G}. \end{array}\right. } \end{aligned}$$

Employing Lemma 4.1, for any $0<\varepsilon _5<<1$, we have

$$\begin{aligned} \limsup _{t\rightarrow \infty } u_1(x,t)< 1-a_1{\underline{s}}_2+a_1\varepsilon _4+\varepsilon _5 \text{ uniformly } \text{ for } x\in \mathcal {G}. \end{aligned}$$

By the arbitrariness of $\varepsilon _4$ and $\varepsilon _5$, it immediately follows that

$$\begin{aligned} \limsup _{t\rightarrow \infty } u_1(x,t)\le 1-a_1{\underline{s}}_2:={\overline{s}}_2 \text{ uniformly } \text{ for } x\in \mathcal {G}. \end{aligned}$$

Hence, for any $0<\varepsilon _5<<1$, there exists $t_5>t_4$ such that

4.24 $$\begin{aligned} u_1(x, t)< {\overline{s}}_2+\varepsilon _5,~ (x, t) \in \mathcal {G}\times [t_5, +\infty ). \end{aligned}$$

(iv) Plugging (4.24) into system (1.7), we find that $u_2$ satisfies

$$\begin{aligned} {\left\{ \begin{array}{ll} \frac{\partial u_2}{\partial t}-d_2\Delta _\omega u_2 \ge u_2(r-a_2{\overline{s}}_2-a_2\varepsilon _5-ru_2), & (x, t)\in \mathcal {G}\times (t_5, +\infty ), \\ u_{2e}(\vartheta ,t)=u_{2e'}(\vartheta ,t), \quad \forall e,e'\sim \vartheta , & (\vartheta , t)\in V\times (t_5, +\infty ),\\ \displaystyle \sum _{e\sim \vartheta }\partial _\nu {u_2}_e(\vartheta ,t)= 0, & (\vartheta , t)\in V\times (t_5, +\infty ),\\ u_2(x, t)|_{t=t_5}=u_2(x, t_5), & x\in \mathcal {G}. \end{array}\right. } \end{aligned}$$

Using Lemma 4.1, for any $0<\varepsilon _6<<1$, we have

$$\begin{aligned} \liminf _{t\rightarrow \infty } u_2(x,t)> 1-\frac{a_2{\overline{s}}_2}{r}-\frac{a_2\varepsilon _5}{r}-\varepsilon _6 \text{ uniformly } \text{ for } x\in \mathcal {G}. \end{aligned}$$

By the arbitrariness of $\varepsilon _5$ and $\varepsilon _6$, we have

4.25 $$\begin{aligned} \liminf _{t\rightarrow \infty } u_2(x,t)\ge 1-\frac{a_2{\overline{s}}_2}{r}:={\underline{s}}_3 \text{ uniformly } \text{ for } x\in \mathcal {G}. \end{aligned}$$

We see that (4.22) and (4.25), (4.21) and (4.24) have the same iterative relations. Therefore, as long as ${\underline{s}}_n\le \frac{1}{a_1}$, we can repeat the above procedure as in (iii) and (iv). In fact, when ${\underline{s}}_n> \frac{1}{a_1}$ the procedure of (i) implies that (4.17) is true. Now it remains to prove the case ${\underline{s}}_n\le \frac{1}{a_1}$. Hence we obtain two sequences $\{{\underline{s}}_n\}$ and $\{{\overline{s}}_n\}$, which satisfy

4.26 $$\begin{aligned} {\underline{s}}_1=\frac{r-a_2}{2r}, ~{\overline{s}}_n=1-a_1{\underline{s}}_n, \text{ and } {\underline{s}}_{n+1}=1-\frac{a_2}{r}{\overline{s}}_n \text{ for } n=1,2\cdots ~ \end{aligned}$$

We now claim that $\{{\underline{s}}_n\}$ is monotone increasing and $\{{\overline{s}}_n\}$ is monotone decreasing. We prove it by using an induction argument. For the case n=1, since $a_2<r$, it is easy to see that

$$\begin{aligned} {\underline{s}}_2-{\underline{s}}_1&=1-\frac{a_2}{r}-\frac{r-a_2}{2r}=\frac{r-a_2}{2r}>0,\\ {\overline{s}}_2-{\overline{s}}_1&=-a_1({\underline{s}}_2-{\underline{s}}_1)<0. \end{aligned}$$

Suppose that ${\underline{s}}_n-{\underline{s}}_{n-1}>0$ and ${\overline{s}}_n-{\overline{s}}_{n-1}<0$. By (4.26), we have

$$\begin{aligned} {\underline{s}}_{n+1}-\underline{s}_n&=-\frac{a_2}{r}({\overline{s}}_{n}-\overline{s}_{n-1})>0,\\ {\overline{s}}_{n+1}-{\overline{s}}_n&=-a_1({\underline{s}}_{n+1}-{\underline{s}}_{n})<0.\end{aligned}$$

Thus, the induction principle yields the claim.

The sequence $\{{\underline{s}}_n\}$ is monotone increasing and the sequence $\{{\overline{s}}_n\}$ is monotone decreasing. Moreover ${\underline{s}}_n\le \frac{1}{a_1}$ and ${\overline{s}}_n\ge 0$, it follows that the limits $\lim _{n\rightarrow \infty }{\underline{s}}_n$ and $\lim _{n\rightarrow \infty }{\overline{s}}_n$ exist, denoted by ${\underline{s}}$ and ${\overline{s}}$, respectively. We now show the claim that ${\underline{s}}_n\le \frac{1}{a_1}$ is not true. By contradiction, assume that ${\underline{s}}_n\le \frac{1}{a_1}$. Then the two sequences $\{{\underline{s}}_n\}$ and $\{{\overline{s}}_n\}$ exist and (4.26) holds. Moreover

4.27 $$\begin{aligned} 0\le {\underline{s}}\le \frac{1}{a_1} \text { and } {\overline{s}}\ge 0. \end{aligned}$$

By letting $n\rightarrow \infty$, (4.26) implies that

$$\begin{aligned} {\left\{ \begin{array}{ll} {\underline{s}}=1-\frac{a_2}{r}{\overline{s}}, \\ {\overline{s}}=1-a_1{\underline{s}}. \end{array}\right. } \end{aligned}$$

Solving the above equations, we have

4.28 $$\begin{aligned} ({\overline{s}}, {\underline{s}})=\left( \frac{r(1-a_1)}{r-a_1a_2}, \frac{r-a_2}{r-a_1a_2} \right) . \end{aligned}$$

Since $a_1>1$, $a_2<r$, (4.28) implies that either ${\overline{s}}<0$ or ${\underline{s}}<0$ holds. In view of (4.27), we have $r-a_1a_2<0$ and $\frac{r-a_2}{r-a_1a_2}>0$, which is a contradiction to the condition $a_2<r$. Thus we have shown the claim ${\underline{s}}_n>\frac{1}{a_1}$.

The procedure of (i) implies that (4.17) is true when $1-a_1{\underline{s}}_n< 0$. Thus we complete the proof. □

We next consider the case that $u_1$ is a superior competitor and $u_2$ is an inferior competitor; namely,

4.29 $$\begin{aligned} a_1<1 \text{ and } a_2>r. \end{aligned}$$

Since the proof of Theorem 4.1 is valid for the case of strong-weak competition, we obtain the following theorem.

#### Theorem 4.2

(Strong-weak competition) Assume that (4.29) holds, then the solution $(u_1, u_2)$ to system (1.7) satisfies

4.30 $$\begin{aligned} \lim _{t\rightarrow \infty }(u_1, u_2)=(1, 0) \text{ uniformly } \text{ for } x\in \mathcal {G}. \end{aligned}$$

### Weak-weak competition

We now examine the case that both $u_1$ and $u_2$ are inferior competitors; namely,

4.31 $$\begin{aligned} a_1<1 \text{ and } a_2<r. \end{aligned}$$

#### Theorem 4.3

(Weak-weak competition) Assume that (4.31) holds, then the solution $(u_1, u_2)$ to system (1.7) satisfies

4.32 $$\begin{aligned} \lim _{t\rightarrow \infty }(u_1, u_2)=\left( \frac{r(1-a_1)}{r-a_1a_2}, \frac{r-a_2}{r-a_1a_2}\right) \text{ uniformly } \text{ for } x\in \mathcal {G}. \end{aligned}$$

#### Proof

By Lemma 3.2, we have $0\le u_1(x, t)\le M_1$ for $(x, t)\in \mathcal {G}\times [0, +\infty )$. Then we find that $u_2$ satisfies

$$\begin{aligned} {\left\{ \begin{array}{ll} \frac{\partial u_2}{\partial t}-d_2\Delta _\omega u_2 \le u_2(r-ru_2), & (x, t)\in \mathcal {G}\times (0, +\infty ), \\ u_{2e}(\vartheta ,t)=u_{2e'}(\vartheta ,t), \quad \forall e,e'\sim \vartheta , & (\vartheta , t)\in V\times (0, +\infty ),\\ \displaystyle \sum _{e\sim \vartheta }\partial _\nu {u_2}_e(\vartheta ,t)= 0, & (\vartheta , t)\in V\times (0, +\infty ),\\ u_2(x, 0)=u_{20}(x)\not \equiv 0, & x\in \mathcal {G}. \end{array}\right. } \end{aligned}$$

Applying Lemma 4.1, we have

$$\begin{aligned} \limsup _{t\rightarrow \infty } u_2(x,t)\le 1:={\overline{v}}_1 \text{ uniformly } \text{ for } x\in \mathcal {G}. \end{aligned}$$

Consequently, for any $0<\varepsilon _1<<1$, there exists $t_1>0$ such that

4.33 $$\begin{aligned} u_2(x, t)< {\overline{v}}_1+\varepsilon _1,&(x, t) \in \mathcal {G}\times [t_1, +\infty ). \end{aligned}$$

(i) Plugging (4.33) into system (1.7), we find that $u_1$ satisfies

$$\begin{aligned} {\left\{ \begin{array}{ll} \frac{\partial u_1}{\partial t}-d_1\Delta _\omega u_1 \ge u_1(1-u_1-a_1{\overline{v}}_1-a_1\varepsilon _1), & (x, t)\in \mathcal {G}\times (t_1, \infty ), \\ u_{1e}(\vartheta ,t)=u_{1e'}(\vartheta ,t), \quad \forall e,e'\sim \vartheta , & (\vartheta , t)\in V\times (t_1, +\infty ),\\ \displaystyle \sum _{e\sim \vartheta }\partial _\nu {u_1}_e(\vartheta ,t)= 0, & (\vartheta , t)\in V\times (t_1, +\infty ),\\ u_1(x, t)|_{t=t_1}=u_1(x, t_1), & x\in \mathcal {G}. \end{array}\right. } \end{aligned}$$

Using Lemma 4.1, for any $0<\varepsilon _2<<1$, there exists $t_2>t_1$ such that

$$\begin{aligned} u_1(x,t)> 1-a_1{\overline{v}}_1-a_1\varepsilon _1-\varepsilon _2,&(x, t) \in \mathcal {G}\times [t_2, +\infty ). \end{aligned}$$

By the arbitrariness of $\varepsilon _1$ and $\varepsilon _2$, we have

$$\begin{aligned} \liminf _{t\rightarrow \infty } u_1(x,t)\ge 1-a_1{\overline{v}}_1:={\underline{w}}_1 \text{ uniformly } \text{ for } x\in \mathcal {G}. \end{aligned}$$

Hence, for any $0<\varepsilon _2<<1$, there exists $t_2>t_1$ such that

4.34 $$\begin{aligned} u_1(x, t)>{\underline{w}}_1-\varepsilon _2,&(x, t) \in \mathcal {G}\times [t_2, +\infty ). \end{aligned}$$

(ii) Substituting (4.34) into system (1.7), we see that $u_2$ satisfies

$$\begin{aligned} {\left\{ \begin{array}{ll} \frac{\partial u_2}{\partial t}-d_2\Delta _\omega u_2\le u_2(r-a_2{\underline{w}}_1+a_2\varepsilon _2-ru_2), & (x, t)\in \mathcal {G}\times (t_2, \infty ), \\ u_{2e}(\vartheta ,t)=u_{2e'}(\vartheta ,t), \quad \forall e,e'\sim \vartheta , & (\vartheta , t)\in V\times (t_2, +\infty ),\\ \displaystyle \sum _{e\sim \vartheta }\partial _\nu {u_2}_e(\vartheta ,t)= 0, & (\vartheta , t)\in V\times (t_2, +\infty ),\\ u_2(x, t)|_{t=t_2}=u_2(x, t_2), & x\in \mathcal {G}. \end{array}\right. } \end{aligned}$$

It follows from Lemma 4.1 that for any $0<\varepsilon _3<<1$, there exists $t_3>t_2$ such that

$$\begin{aligned} u_2(x,t)< 1-\frac{a_2}{r}{\underline{w}}_1+\frac{a_2}{r}\varepsilon _2+\varepsilon _3,&(x, t) \in \mathcal {G}\times [t_3, +\infty ). \end{aligned}$$

The arbitrariness of $\varepsilon _2$ and $\varepsilon _3$ implies that

$$\begin{aligned} \limsup _{t\rightarrow \infty } u_2(x,t)\le 1-\frac{a_2}{r}{\underline{w}}_1:={\overline{v}}_2 \text{ uniformly } \text{ for } x\in \mathcal {G}. \end{aligned}$$

Consequently, for any $0<\varepsilon _3<<1$, there exists $t_3>t_2$ such that

4.35 $$\begin{aligned} u_2(x, t)<{\overline{v}}_2+\varepsilon _3 ,&(x, t) \in \mathcal {G}\times [t_3, +\infty ). \end{aligned}$$

(iii) Plugging (4.35) into system (1.7), we obtain that $u_1$ satisfies

$$\begin{aligned} {\left\{ \begin{array}{ll} \frac{\partial u_1}{\partial t}-d_1\Delta _\omega u_1 \ge u_1(1-u_1-a_1{\overline{v}}_2-a_1\varepsilon _3), & (x, t)\in \mathcal {G}\times (t_3, \infty ), \\ u_{1e}(\vartheta ,t)=u_{1e'}(\vartheta ,t), \quad \forall e,e'\sim \vartheta , & (\vartheta , t)\in V\times (t_3, +\infty ),\\ \displaystyle \sum _{e\sim \vartheta }\partial _\nu {u_1}_e(\vartheta ,t)= 0, & (\vartheta , t)\in V\times (t_3, +\infty ),\\ u_1(x, t)|_{t=t_3}=u_1(x, t_3), & x\in \mathcal {G}. \end{array}\right. } \end{aligned}$$

Once again Lemma 4.1 implies that for any $0<\varepsilon _4<<1$, there exists $t_4>t_3$ such that

$$\begin{aligned} u_1(x,t)> 1-a_1{\overline{v}}_2-a_1\varepsilon _3-\varepsilon _4,&(x, t) \in \mathcal {G}\times [t_4, +\infty ). \end{aligned}$$

By the arbitrariness of $\varepsilon _3$ and $\varepsilon _4$, one has

$$\begin{aligned} \liminf _{t\rightarrow \infty } u_1(x,t)\ge 1-a_1{\overline{v}}_2:={\underline{w}}_2 \text{ uniformly } \text{ for } x\in \mathcal {G}. \end{aligned}$$

Thus, for any $0<\varepsilon _4<<1$, there exists $t_4>t_3$ such that

4.36 $$\begin{aligned} u_1(x, t)>{\underline{w}}_2-\varepsilon _4,&(x, t) \in \mathcal {G}\times [t_4, +\infty ). \end{aligned}$$

(iv) Substituting (4.36) into system (1.7) yields that $u_2$ satisfies

$$\begin{aligned} {\left\{ \begin{array}{ll} \frac{\partial u_2}{\partial t}-d_2\Delta _\omega u_2\le u_2(r-a_2{\underline{w}}_2+a_2\varepsilon _4-ru_2), & (x, t)\in \mathcal {G}\times (t_4, \infty ), \\ u_{2e}(\vartheta ,t)=u_{2e'}(\vartheta ,t), \quad \forall e,e'\sim \vartheta , & (\vartheta , t)\in V\times (t_4, +\infty ),\\ \displaystyle \sum _{e\sim \vartheta }\partial _\nu {u_2}_e(\vartheta ,t)= 0, & (\vartheta , t)\in V\times (t_4, +\infty ),\\ u_2(x, t)|_{t=t_4}=u_2(x, t_4), & x\in \mathcal {G}. \end{array}\right. } \end{aligned}$$

Using Lemma 4.1 one more time, for any $0<\varepsilon _5<<1$, there exists $t_5>t_4$ such that

$$\begin{aligned} u_2(x,t)< 1-\frac{a_2}{r}{\underline{w}}_2+\frac{a_2}{r}\varepsilon _4+\varepsilon _5,&(x, t) \in \mathcal {G}\times [t_5, +\infty ). \end{aligned}$$

It follows from the arbitrariness of $\varepsilon _4$ and $\varepsilon _5$ that

$$\begin{aligned} \limsup _{t\rightarrow \infty } u_2(x,t)\le 1-\frac{a_2}{r}{\underline{w}}_2:={\overline{v}}_3 \text{ uniformly } \text{ for } x\in \mathcal {G}. \end{aligned}$$

Therefore, (4.33) and (4.35), (4.34) and (4.36) have the same iterative relation. Consequently, if the sequence $\{{\underline{w}}_n\}$ is monotone increasing and the sequence $\{{\overline{v}}_n\}$ is monotone decreasing, then condition (4.2) is satisfied. We can apply Lemma 4.1 once again and repeat the above procedure as in (i), (ii), (iii) and (iv) to obtain two sequences $\{{\underline{w}}_n\}$ and $\{{\overline{v}}_n\},$ which satisfy

4.37 $$\begin{aligned} {\overline{v}}_1=1, ~{\underline{w}}_n=1-a_1{\overline{v}}_n, \text{ and } {\overline{v}}_{n+1}=1-\frac{a_2}{r}{\underline{w}}_n, \text{ for } n=1,2\cdots \end{aligned}$$

Similarly, we can prove that $\{{\underline{w}}_n\}$ is monotone increasing and $\{{\overline{v}}_n\}$ is monotone decreasing by using induction. For the case n=1, since $a_1<1$, it is easy to see that

$$\begin{aligned}{\left\{ \begin{array}{ll} {\overline{v}}_2-{\overline{v}}_1=-\frac{a_2}{r}{\underline{w}}_1=-\frac{a_2}{r}(1-a_1)<0,\\ {\underline{w}}_2-{\underline{w}}_1=-a_1({\overline{v}}_2-{\overline{v}}_1)>0. \end{array}\right. }\end{aligned}$$

Suppose that ${\overline{v}}_n-{\overline{v}}_{n-1}<0, ~\underline{w}_n-{\underline{w}}_{n-1}>0$. By (4.37), we have

$$\begin{aligned} {\left\{ \begin{array}{ll} {\overline{v}}_{n+1}-{\overline{v}}_n=1-\frac{a_2}{r}\underline{w}_n-(1-\frac{a_2}{r}{\underline{w}}_{n-1})=-\frac{a_2}{r}(\underline{w}_n-{\underline{w}}_{n-1})<0,\\ {\underline{w}}_{n+1}-\underline{w}_n=-a_1({\overline{v}}_{n+1}-{\overline{v}}_n)>0. \end{array}\right. }\end{aligned}$$

By induction, we obtain the conclusion on the monotonicity of $\{{\underline{w}}_n\}$ and $\{{\overline{v}}_n\}$.

Consequently, the limits $\lim _{n\rightarrow \infty }{\underline{w}}_n$ and $\lim _{n\rightarrow \infty }{\overline{v}}_n$ exist and are denoted by ${\underline{w}}$ and ${\overline{v}}$, respectively. Hence, (4.37) implies that

$$\begin{aligned} {\left\{ \begin{array}{ll} {\overline{v}}=1-\frac{a_2}{r}{\underline{w}}, \\ {\underline{w}}=1-a_1{\overline{v}}. \end{array}\right. } \end{aligned}$$

Solving the above equations, we have ${\underline{w}}=\frac{r(1-a_1)}{r-a_1a_2}$ and ${\overline{v}}=\frac{r-a_2}{r-a_1a_2}$. Therefore, the above argument shows that

4.38 $$\begin{aligned} {\left\{ \begin{array}{ll} \liminf _{t\rightarrow \infty } u_1\ge {\underline{w}}=\frac{r(1-a_1)}{r-a_1a_2} \text { uniformly for } \mathcal {G}, \\ \limsup _{t\rightarrow \infty } u_2\le \overline{v}=\frac{r-a_2}{r-a_1a_2} \text { uniformly for } \mathcal {G}. \end{array}\right. } \end{aligned}$$

In a similar way, we can induce that

4.39 $$\begin{aligned} {\left\{ \begin{array}{ll} \limsup _{t\rightarrow \infty } u_1\le \frac{r(1-a_1)}{r-a_1a_2} \text { uniformly for } \mathcal {G}, \\ \liminf _{t\rightarrow \infty } u_2\ge \frac{r-a_2}{r-a_1a_2} \text { uniformly for } \mathcal {G}. \end{array}\right. } \end{aligned}$$

Hence we have

$$\begin{aligned} {\left\{ \begin{array}{ll}\lim _{t\rightarrow \infty } u_1=\frac{r(1-a_1)}{r-a_1a_2} \text { uniformly for } \mathcal {G}, \\ \lim _{t\rightarrow \infty } u_2=\frac{r-a_2}{r-a_1a_2} \text { uniformly for } \mathcal {G}. \end{array}\right. }\end{aligned}$$

This completes the proof. □

## Numerical simulations: Invasion and competition of *Ae. aegypti* and *Ae. albopictus* in Florida

We aim to simulate the competition between the invasive *Ae. albopictus* and native *Ae. aegypti* via State Route 441 in Florida (Figure 1). We only consider 11 cities with relative size, Lake City, Gainesville, Ocala, Leesburg, Apopka, Orlando, Kissimmee, St. Cloud, Yeehaw Junction, Okeechobee, and Miami along Route 441 as the vertices of a graph $\mathcal {G}$, while every path linking these 11 cities of Route 441 are the edges of $\mathcal {G}$. We regard these 11 cities as vertices and connect two vertices by an edge only if the corresponding cities are consecutive along Route 441 in our dataset. Hence the resulting graph is a path graph (vertex degree ≤2). We emphasize that the graph used in the numerical simulations is a compact metric graph with degree at most two at each vertex, corresponding to a linear chain along Florida State Route 441. In particular, the graph is not a complete graph, despite the geographical visualization of the cities along the route.

Verdonschot and Besse-Lototskaya (2014) reported that the average flight distances of *Ae. aegypti* and *Ae. albopictus* are 333m (standard deviation 384m) and 676m (standard deviation 458m), respectively. So it is biologically reasonable to choose $D_1=0.0125$km and $D_2=0.025$km, which are in agreement with the short dispersal experiments and field studies (Oteroa et al. 2008). The other parameters are also chosen in a significant scope such as in Takahashi et al. (2005). For system (1.7), we consider the values for the dimensional parameters in the following unit system (*space* = [*x*] = km, *time* = [*t*] = day):

5.1 $$\begin{aligned} \begin{aligned}&D_1=0.0125, D_2=0.025, r_1=0.02, r_2=0.02,\\ &K_1=25, K_2=25, \tilde{a}_1,~\tilde{a}_2\in [2.5\times 10^{-5},~ 8\times 10^{-4}]. \end{aligned}\end{aligned}$$

**Fig. 2:**
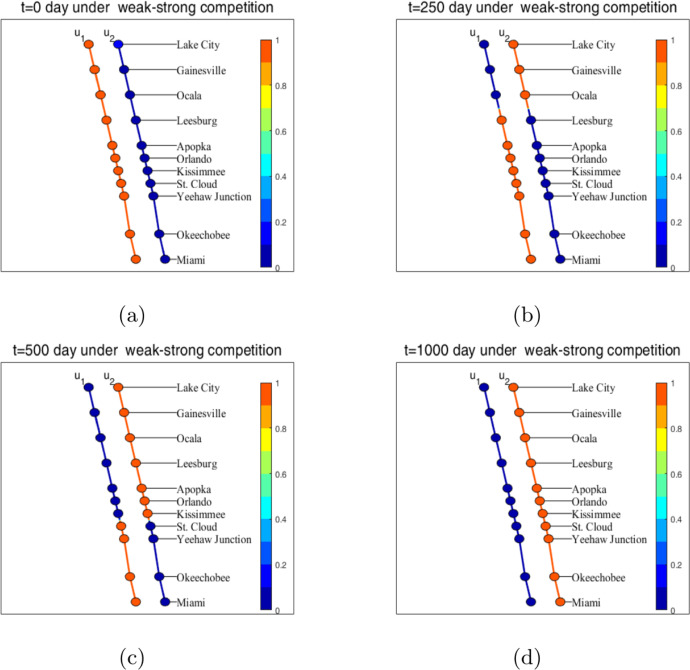
The long time behavior of $u_1$ (*Ae. aegypti*) and $u_2$ (*Ae. albopictus*) of system (1.7) for the weak-strong competition case. The network $\mathcal {G}$ is simulated by Route 441 in Florida. The initial values are $u_1=1$ in the whole Route 441, $u_2=0.1$ only on the habitat Lake City and $u_2=0$ on the other cities and whole Route 441

In view of Figure 1, we find that there are three competition outcomes in Florida: in North Florida, such as Lake City and Gainesville, with *Ae. albopictus* only; in Central Florida, such as Apopka and Orlando, coexistence of *Ae. aegypti* and *Ae. albopictus*; and in South Florida, such as Miami, with *Ae. aegypti* dominating. Moreover, Hopperstad and Reiskind (2016) reported that the interaction between *Ae. aegypti* and *Ae. albopictus* may be attenuated through natural selection. Hence the strength of interactions between *Ae. aegypti* and *Ae. albopictus* may be different in different cities. We present four possible competitive interaction strengths to explain the invasion and competition of *Ae. aegypti* and *Ae. albopictus* in Florida.

**(i) Weak-strong competition.** In this case, we assume that the non-dimensional parameters take the following values

5.2 $$\begin{aligned} d_1=0.625, d_2=1.25, r=1, a_1=1.2, a_2=0.08. \end{aligned}$$

It follows from (5.2) that *Ae. aegypti* and *Ae. albopictus* are weak-strong competitors. Theorem 4.1 implies that in this case only *Ae. albopictus* is survival. In Figure 2, the initial value is that $u_1=1$ in the whole Route 441, $u_2=0.1$ only on the habitat Lake City and $u_2=0$ on the other cities and whole Route 441. This is used to simulate the scenario that *Ae. albopictus* was firstly introduced in northern cities in Florida in 1986 and then spread southward along Route 441 to the whole Florida state in 1994 (O’meara et al. 1995).

Figure 2 shows that the invasion of *Ae. albopictus* along Route 441. First *Ae. albopictus* ($u_2$) spreads out in North Florida in Figure 2 (b), then *Ae. albopictus* expands to Central Florida in Figure 2 (c), eventually *Ae. albopictus* spreads to South Florida in Figure 2 (d). Moreover, when *Ae. albopictus* ($u_2$) spreads out, the domestic *Ae. aegypti* ($u_1$) goes extinct. This phenomenon is agreement with the observation by O’Meara et al. (1992) that at the beginning major declines in *Ae. aegypti* abundance in Florida were believed to be associated with the invasion and expansion of *Ae. albopictus* populations.

**Fig. 3:**
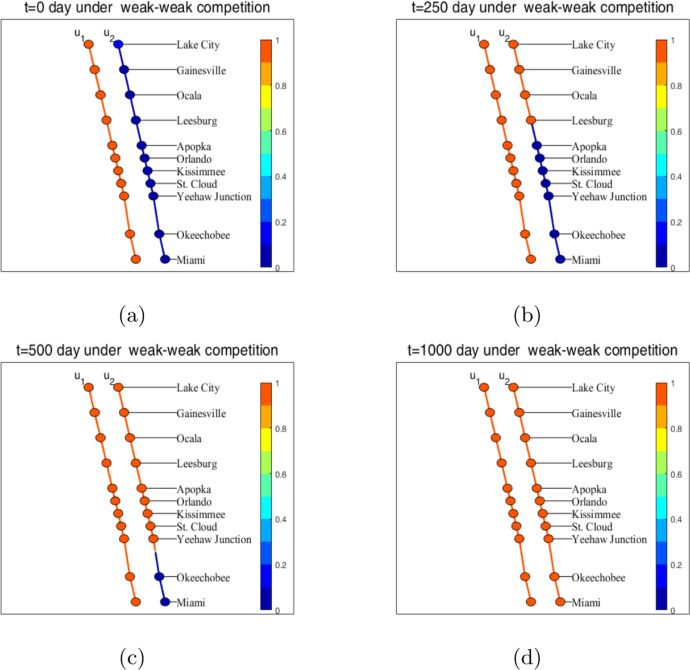
The long time behavior of $u_1$ (*Ae. aegypti*) and $u_2$ (*Ae. albopictus*) of system (1.7) for the weak-weak competition case. The network $\mathcal {G}$ is simulated by Route 441 in Florida. The initial values are that $u_1=1$ in the whole Route 441, $u_2=0.1$ only on the habitat Lake City and $u_2=0$ on the other cities and whole Route 441

**(ii) Weak-weak competition.**Parker et al. (2019) reported that since 1995 *Ae. aegypti* reappeared again in the Central Florida where it was previously displaced by *Ae. albopictus*. Moreover, the habitat of *Ae. aegypti* is expanding. This phenomenon implies that the strength of interaction between *Ae. aegypti* and *Ae. albopictus* may change due to natural selection (Hopperstad and Reiskind 2016). Hence we assume that the non-dimensional parameters as follows

5.3 $$\begin{aligned} d_1=0.625, d_2=1.25, r=1, a_1=0.05, a_2=0.08. \end{aligned}$$

so that *Ae. aegypti* and *Ae. albopictus* are weak-weak competitors. Theorem 4.3 implies that in this case *Ae. aegypti* and *Ae. albopictus* coexist and the numerical simulations are shown in Figure 3 . This simulates the scenario that since 1995 *Ae. aegypti* ($u_1$) reappeared again in central cities of Florida along Route 441; meanwhile *Ae. albopictus* ($u_2$) also survives in central cities of Florida. Nowadays the habitat of *Ae. aegypti* is expanding, which means that mosquitoes’ behavior belongs to the stage of Figure 3 (c). In Figure 3 (c), the wave front locates between Yeehaw Junction and Okeechobee. A superior competition *Ae. aegypti* equipped with a slow diffusion exhibits a traveling wave where the wave front can be observed by the state of *Ae. albopictus*.

**Fig. 4:**
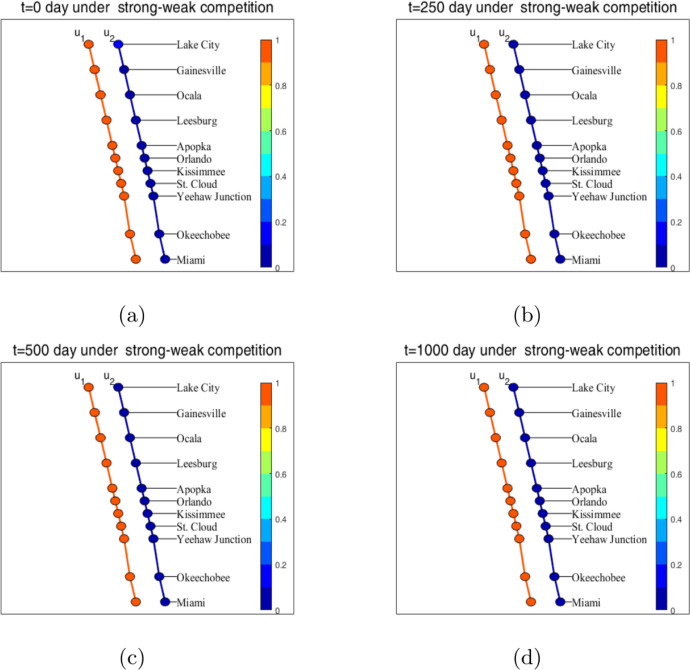
The long time behavior of $u_1$ (*Ae. aegypti*) and $u_2$ (*Ae. albopictus*) of system (1.7) for the strong-weak competition case. The network $\mathcal {G}$ is simulated by State Route 441 in Florida. The initial values are $u_1=1$ in the whole Route 441, $u_2=0.1$ only on the habitat Lake City and $u_2=0$ on the other cities and whole Route 441

**(iii) Strong-weak competition.** In both 1994 and 2014 (Figure 1(a)), it was observed that in Miami the native *Ae. aegypti* is dominating. In this case, we assume that the non-dimensional parameters are such as

5.4 $$\begin{aligned} d_1=0.625, d_2=1.25, r=1, a_1=0.05, a_2=1.2. \end{aligned}$$

Then *Ae. aegypti* and *Ae. albopictus* are strong-weak competitors such as in Miami. Theorem 4.2 implies that in this case only *Ae. aegypti* is surviving in the long term. Figure 4 shows that the native *Ae. aegypti* ($u_1$) spreads along Route 441, meanwhile the invasive *Ae. albopictus* ($u_2$) is vanishing along Route 441.

## Discussions

In Florida, *Ae. aegypti* is a local mosquito species and *Ae. albopictus* was introduced in North Florida in 1986 and is an invasive mosquito species. It has been reported that *Ae. albopictus* competes with *Ae. aegypti* through satyrization, larval competition, and climatic differences in Florida (Parker et al. 2019). Due to its invasion ability and its competition with *Ae. aegypti*, *Ae. albopictus* had been reported in the whole state of Florida by 1995. Our competition model with compact metric graph (1.7) can be applied to describe the invasion of *Ae. albopictus* and the competition dynamics between these two species.

While the mechanisms underlying biological invasion remain incompletely understood, researchers have increasingly employed diverse population dynamics models to explore this phenomenon. Mathematical frameworks such as reaction-diffusion equations (Aronson and Weinberger 1975, 1978; Fisher 1937; Liang and Zhao 2007; Skellam 1951; Weinberger et al. 2007) and heterogeneous environment models (Lou 2006; He and Ni 2016, and Zhang et al. 2017) have been used to investigate species spreading through analyses of traveling wave solutions. These studies typically utilize Laplacian operators to characterize population dispersal, under the assumption that individuals follow a Gaussian random walk principle, i.e., equal probability of movement in all directions. However, in Florida *Ae. albopictus* has expanded its geographic range through expressways. Hence our competition model in a compact metric graph differs from these approaches: the movement of mosquitoes at each graph vertex is explicitly governed by the topological structure of the graph. We analyze the long-term dynamical behavior of the invasive *Ae. albopictus* competing with *Ae. aegypti*. To the best of our knowledge, no existing population dynamics model in the literature has incorporated compact metric graph to characterize biological invasions.

Using our model, we can partially explain the mechanism of invasion and competition of *Aedes* mosquitoes in Florida. In the scenario of weak-strong competition, Theorem 4.1 shows that *Ae. albopictus* outcompetes the domestic *Ae. aegypti*. This is consistent with the field observation that *Ae. albopictus* had been found in the entire state from 1986 to 1995 (Figure 1 (a)), and that in the Panhandle region *Ae. albopictus* was in present exclusively with no *Ae. aegypti* in 2019 (Figure 1 (b)). However, the competitive ability of *Ae. aegypti* may become strong due to natural selection. The weak-strong competition may change to weak-weak competition. In this scenario Theorem 4.3 shows that *Ae. aegypti* and *Ae. albopictus* coexist in the competition. This is consistent with the report that *Ae. aegypti* and *Ae. albopictus* coexisted in Central Florida in 2019. In South Florida, both *Ae. aegypti* and *Ae. albopictus* were present while *Ae. aegypti* is dominating in 2019 (Figure 1 (b)), which indicates that it was at certain stage of strong-weak competition. Moreover, our numerical simulations reveal that the evolution process induces traveling waves (Figure 2 (b)). As a result, we can make early warnings and formulate response strategies for mosquito spreading based on the speed of traveling wave solutions, so as to reduce the risk of mosquito-borne diseases.

## Data Availability

This paper has no associated data.
